# Revision Arthroplasty Through the Direct Anterior Approach Using an Asymmetric Acetabular Component

**DOI:** 10.3390/jcm9093031

**Published:** 2020-09-21

**Authors:** Peter Michael Prodinger, Igor Lazic, Konstantin Horas, Rainer Burgkart, Rüdiger von Eisenhart-Rothe, Manuel Weissenberger, Maximilian Rudert, Boris Michael Holzapfel

**Affiliations:** 1 Department of Orthopaedic Surgery, Krankenhaus Agatharied, Norbert-Kerkel-Platz, 83734 Hausham, Germany; peter.prodinger@khagatharied.de; 2 Department of Orthopaedic Surgery, Klinikum Rechts der Isar, Technical University Munich, Ismaninger Str. 22, 81675 Munich, Germany; igr.lzc@googlemail.com (I.L.); rainer.burgkart@tum.de (R.B.); eisenhart@tum.de (R.v.E.-R.); 3 Department of Orthopaedic Surgery, University of Wuerzburg, Koenig-Ludwig-Haus, Brettreichstrasse 11, 97074 Wuerzburg, Germany; k-horas.klh@uni-wuerzburg.de (K.H.); m-weissenberger.klh@uni-wuerzburg.de (M.W.); m-rudert.klh@uni-wuerzburg.de (M.R.); 4 Regenerative Medicine, Institute of Health and Biomedical Innovation, Queensland University of Technology (QUT), 60 Musk Avenue, Kelvin Grove, Brisbane, QLD 4059, Australia

**Keywords:** anterior approach, revision arthroplasty, hip joint, acetabular bone defect, asymmetric implant, anatomic center of rotation

## Abstract

Despite increasing numbers of primary hip arthroplasties performed through the direct anterior approach (DAA), there is a lack of literature on DAA revision arthroplasty. The present study was performed in order to evaluate outcomes and revision rates after revision through the DAA using an asymmetric acetabular component with optional intra- and extramedullary fixation. In a retrospective cohort study, we analyzed prospectively collected data of 57 patients (61 hips, 43 female, 18 male) who underwent aseptic acetabular component revision through the DAA with the abovementioned implant system between January 2015 and December 2017. The mean follow-up was 40 months (12–56). Survival rates were estimated using the Kaplan–Meier method. All complications were documented and functional outcomes were assessed pre- and postoperatively. Kaplan–Meier analysis revealed an estimated five-year implant survival of 97% (confidence interval CI 87–99%). The estimated five-year survival with revision for any cause was 93% (CI 83–98%). The overall revision rate was 6.6% (n = 4). Two patients had to undergo revision due to periprosthetic infection (3.3%). In one patient, the acetabular component was revised due to aseptic loosening four months postoperatively. Another patient suffered from postoperative iliopsoas impingement and was treated successfully by arthroscopic iliopsoas tenotomy. Two (3.3%) of the revised hips dislocated postoperatively. The mean Harris Hip Score improved from 35 (2–66) preoperatively to 86 (38–100) postoperatively (*p* < 0.001). The hip joint’s anatomical center of rotation was restored at a high degree of accuracy. Our findings demonstrate that acetabular revision arthroplasty through the DAA using an asymmetric acetabular component with optional intra- and extramedullary fixation is safe and practicable, resulting in good radiographic and clinical midterm results.

## 1. Introduction

In the direct anterior approach (DAA), the surgeon enters the hip joint through the intermuscular and interneural plane between the tensor fasciae latae (TFL) and the sartorius muscle [1]. In comparison to lateral approaches, the DAA is supposed to be associated with an accelerated early functional recovery and a reduced rate of postoperative Trendelenburg-positive patients [2]. Recent analyses of arthroplasty registries demonstrated that the DAA is further associated with a lower risk of revision for dislocation when compared to posterior approaches [3,4,5]. Moreover, due to the supine positioning of DAA patients, intraoperative leg length evaluation and fluoroscopic control of implant positioning may be facilitated. These advantages, among other reasons, may have led to an increasing popularity of the DAA in Europe and the U.S. [6,7]. Recently, an American Association of Hip and Knee Surgeons (AAHKS) survey documented an increasing use of the DAA among respondents, from 12% in 2010 to 40% in 2018 [6]. 

Parallel to the increasing number of hip procedures performed via the DAA, the number of patients with clinical problems after DAA hip arthroplasty will inevitably increase [6]. Hip surgeons and their patients have to be aware of the disadvantages associated with the use of the DAA, such as a steep learning curve and a putatively higher infection rate when compared to other approaches [8,9,10]. Moreover, questions have been raised about the practicability of hip revisions through the DAA. According to a recent AAHKS survey, some respondents still answer with “because the approach is not extensile” when asked about the single, most important reason why they are not performing the DAA [7]. 

Recent clinical and anatomical studies however have demonstrated that revision arthroplasty through the DAA is indeed feasible and clinical and radiographic outcomes after femoral or acetabular revision can be comparable to outcomes after hip revision through other approaches [11,12,13,14,15]. Surgeons performing hip revision arthroplasty via the DAA do not only have to know the anatomy of the Hueter interval and its surrounding structures in detail but they also have to be familiar with the specific instruments needed for successful implant fixation. Ideally, the revision implant used is not only suitable to treat any kind of osseous defect but also to be handled through the DAA without the need for extensive tissue releases [16]. 

The purpose of the following study was to analyze the clinical and radiographic outcome of patients who underwent aseptic acetabular component revision through the DAA using an asymmetric acetabular component with optional extra- and intramedullary iliac fixation.

## 2. Materials and Methods

We performed a noncomparative, retrospective cohort study including all patients who underwent aseptic acetabular component revision through the DAA with the abovementioned implant system between January 2015 and December 2017 (n = 64 patients). Institutional review board approval was obtained prior to data analysis (no. 2018012601, University of Wuerzburg). Data for this study was prospectively collected by our institution’s arthroplasty study unit. Patients were excluded if they were younger than 18 years or if they had been treated due to pelvic tumor. A minimum follow-up of 12 months was mandatory. This left 57 patients (18 male, 43 female, 61 hips) with a mean follow-up of 40 months (12–56) to be included in the study. Reasons for revision were aseptic loosening and periacetabular osseous defects in 58 hips (three with chronic pelvic discontinuity, two with acute pelvic discontinuity and medial cup protrusion), metal-on-metal pseudotumor in two hips, and recurrent hip dislocation in one hip. The mean age at the time of surgery was 69 years (42–93) and the mean body mass index was 26 kg/m^2^ (17–36). 

In 70% (n = 43) of the hips, this was the first revision, whereas 30% (n = 18) of the hips underwent at least one previous revision (1–11). In 14 hips, the index procedure was performed in the authors’ department, while all others were referrals. The mean time between the index surgery and the revision under observation was 11 years (0–23). From the 43 hips that underwent no previous revision, seven were implanted via the DAA, 21 via the direct lateral, and 15 via the posterior approach. From the 18 hips that underwent at least one previous revision, 11 were previously revised via the direct lateral and five via the posterior approach. Two patients were previously revised multiple times through different approaches with crossing scars. 

**Figure 1:**
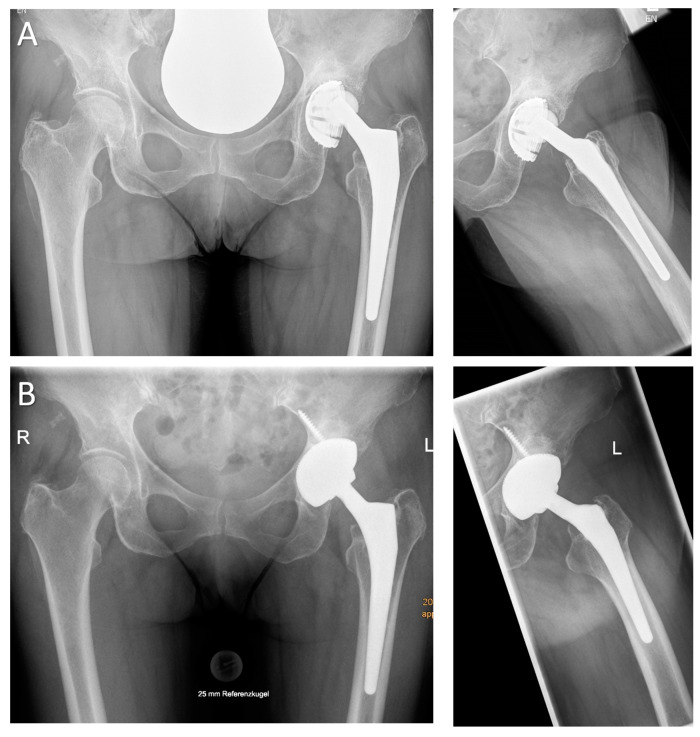
(**A**) A 72-year-old female patient presenting with significant polyethylene wear and aseptic loosening of the acetabular component which was implanted 11 years ago (antero-posterior and axial views). The intraoperative bone defect evaluation revealed a cranial and lateral segmental defect with supportive dome and acetabular rim (Paprosky type IIB). (**B**) Radiographic imaging 12 months postoperatively (a.p. and axial views) demonstrate a well-fixed asymmetric acetabular component filling the cranial defect. The distal positioning of the insert makes it possible to reconstruct the anatomical center of rotation and to treat any pre-existing leg-length discrepancy (HHS: 56 preoperatively, 91 postoperatively; operation time OT 72 min).

All patients included in this study underwent revision surgery through the DAA without the use of a traction table. Patients were operated in supine position and the hip joint was exposed through the Hueter interval, as previously described [1,17,18,19]. Then, the neo-capsule was carefully resected. After dislocation of the hip, the femoral head and the loose acetabular implant were removed. In three patients, the well-fixed acetabular implant had to be removed due to pseudotumor and recurrent dislocation, respectively. Periacetabular debris and membranes were removed using sharp curettes and a small acetabular reamer. Although we graded the osseous defects radiographically using the Paprosky classification scheme, the definitive classification was performed intraoperatively [16,20]. Using the Paprosky classification scheme, we classified 28 bone defects as IIB, 10 as IIC, 12 as IIIA, and six as IIIB. Furthermore, there were two patients with periacetabular fracture and acute pelvic discontinuity and three patients with IIIB defects and concomitant chronic pelvic discontinuity. In 51 cases, we performed an isolated acetabular component revision, whereas in 10 cases the femoral stem had to be revised as well. For acetabular revision, the standard approach was used without any additional muscular releases. Acetabular reaming was performed in a standardized manner. First, we reamed as caudally as possible with ascending reamer sizes. With the last reamer in place, the location and the dimensions of the cranial bone defect was determined. The trial cup and the definitive implant are both asymmetric, exhibiting two overlapping radii with the cranial one being 6 mm smaller than the caudal one. The polyethylene insert is positioned at the center of the caudal radius to restore the anatomical center of rotation (Figure 1). 

**Figure 2:**
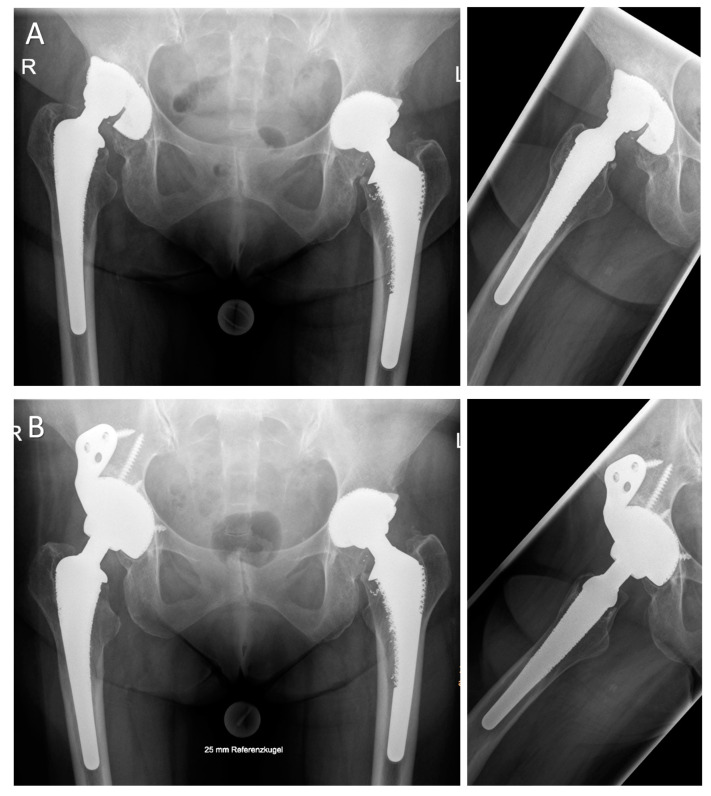
(**A**) A 63-year-old female patient with failure of the acetabular implant and subsequent cranial migration 18 years after the index procedure (a.p. and axial views). Intraoperative defect evaluation revealed a large uncontained cranio-lateral defect (“up-and-out”) with rim destruction of more than 30% (Paprosky type IIIA defect). (**B**) Radiographs 24 months postoperatively revealed a stable acetabular implant augmented with multiple screws through the implant’s body and the iliac flange (HHS: 25 preoperatively, 81 postoperatively; OT 79 min).

Position and fit of the trial component are tested and controlled fluoroscopically. The acetabular component is available in a standard version and one version in which the implant’s body carries an anatomic iliac flange in a mono-block fashion to provide additional stability in the presence of significant medial wall defects (protrusion) or rim destruction of more than 30% [21,22] (Figure 2).

**Figure 3:**
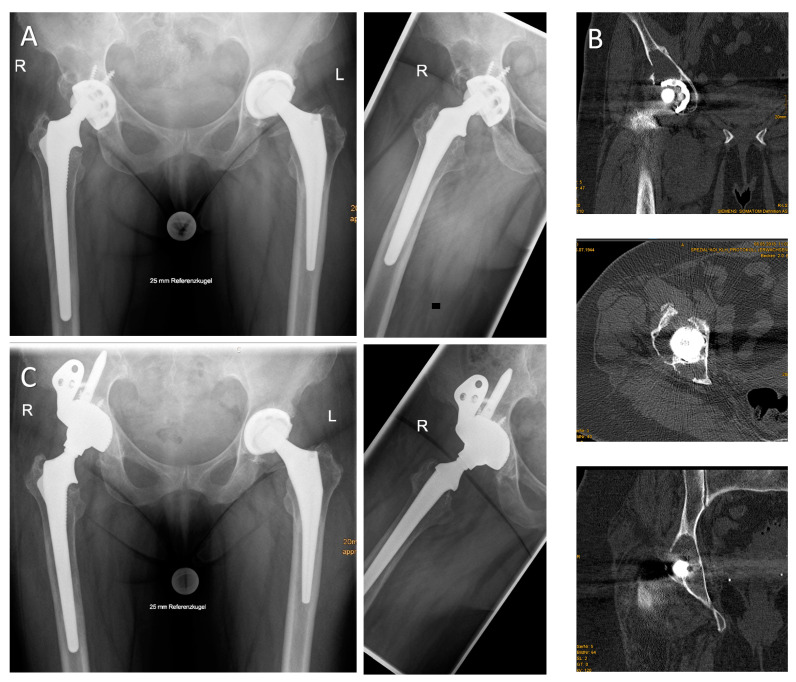
(**A**) A.p. and axial radiographs of an 81-year-old female patient with large periacetabular osteolyses due to wear debris 17 years after primary hip arthroplasty. (**B**) Multiaxial CT analyses revealed insufficient stability of both the anterior and posterior column. Intraoperatively, the large iliac defect was contained by an egg shell-like outer cortex. More than 60% of the acetabular rim was deficient. The iliac defect was filled with impacted allogenic bone chips and the asymmetric cup was augmented with a 50-mm intramedullary iliac press-fit stem and multiple screws. (**C**) Radiographic imaging 24 months postoperatively revealed a stable implant with well-integrated bone grafts (HHS: 46 preoperatively, 66 postoperatively; OT 150 min).

In our study, 21 patients received a standard implant whereas 40 patients received a component armed with an iliac flange. The screw fixation through the implant’s iliac flange can be performed by insertion of the screws via the DAA and subsequent fixation with a long screwdriver introduced via a gluteal stab incision. In defects with rim destruction of more than 60% or in defects with pelvic discontinuity, the implant can be augmented with an intramedullary iliac press-fit stem (10.5 mm; length 30, 50, or 70 mm) in order to further reduce shear forces that may act on the cup (Figure 3). 

**Figure 4:**
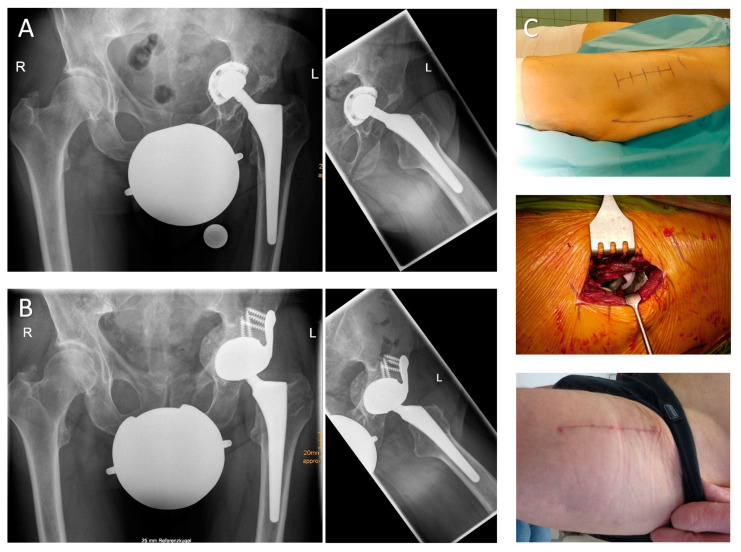
(**A**) A.p. and axial radiograph of a 66-year-old male patient with significant medial cup protrusion. The intraoperative defect classification revealed an intact anterior and posterior column with supportive rim elements (Paprosky type IIC). (**B**) The defect was filled with impacted allogeneic bone chips obtained from two femoral heads. A.p. and axial radiographs 12 months postoperatively demonstrate sufficient integration and remodeling of the graft and reconstruction of the anatomical center of rotation. (**C**) Pre-, intra-, and postoperative views showing the skin incision used for DAA revision arthroplasty and its position relative to the previously used approach (HHS: 66 preoperatively, 100 postoperatively; OT 149 min).

An additional intramedullary stem was implanted in 14 of our patients. The acetabular component used (Revisio M, AQ solutions, Germany) is coated with hexapodal structures (Spongiosa metal II^®^) to promote osseo-integration. The largest available cranio-caudal diameter of the cup is 82 mm. Prior to definitive component placement, contained defects can be filled with bone allografts or ceramic bone substitute materials, if deemed necessary. In this study, we implanted allogenic bone chips in 10 and ceramic particles in 14 cases (Figure 4). 

The definitive implant is impacted in a size 2 mm larger than the last reamer to achieve a certain degree of press-fit. Additional screw fixation can be performed through the implant’s body and the flange with 6.5-mm self-tapping cancellous screws. The mean number of screws used in our study population was three (1–5). The head diameter used was 28 mm in nine, 32 mm in 32, and 36 mm in 20 patients. In 13 patients, an asymmetric liner was used with a 10° elevated lip and all other patients received standard polyethylene liners. In 51 patients, we performed an isolated cup revision. The mean operation time (OT) in these patients was 113 min (46–218). 

**Figure 5:**
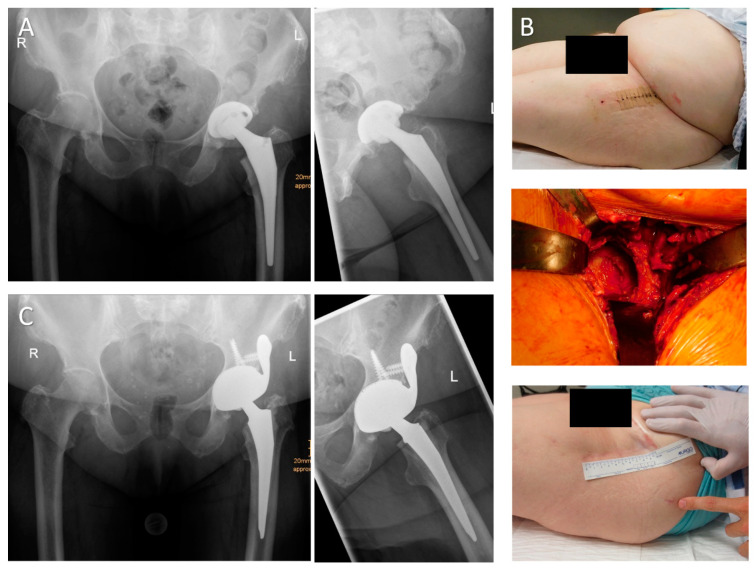
(**A**) A.p. and axial radiograph of an 80-year-old female patient presenting with transverse periprosthetic fracture and medial protrusion of the cup two weeks after cementless hip arthroplasty. (**B**) The patient suffered from multiple co-morbidities and her BMI (body mass index) was 39 kg/m^2^. After removal of the loose acetabular and femoral implant via the DAA, the acetabular fracture line was visible. To prevent iatrogenic lesions of the TFL during stem extraction, a “tensor-snip” was performed. The screw fixation through the implant’s iliac flange was performed by insertion of the screws via the DAA and subsequent fixation with a long screwdriver introduced via a gluteal stab incision in order to avoid any proximal extension of the approach. (**C**) Radiographic imaging (a.p. and axial view) 18 months postoperatively show a well-fixed acetabular implant (HHS: nine preoperatively, 64 postoperatively; OT 126 min).

In 10 patients, we revised both the acetabular and femoral component. Here, the mean operation time was 158 min (90–230). To prevent iatrogenic lesions of the TFL muscle during stem removal, a “tensor snip” was performed in these cases (Figure 5). Therefore, half the width of the TFL tendon is cut from medially to laterally 1 cm distal to its origin at the anterior part of the external lip of the iliac crest, holding the affected leg in adduction and internal rotation to pretension the muscle. The surgeon is then able to feel an instantaneous release and the TFL can be mobilized laterally to provide access to the femoral canal. As the attachment of the TFL at the gluteal aponeurosis stays intact, the TFL remains enveloped within the layers of the fascia lata posteriorly and distally [23]. Therefore, at the end of surgery, closure of the deep and superficial layer of the fasciae latae is enough to ensure proper healing. After performing the “tensor snip”, there is no need to delay postoperative rehabilitation. For further exposure of the joint, no other additional muscular releases are necessary. All patients in this study were allowed full weight bearing from the first day after surgery.

Clinical outcome assessment was performed using the Harris Hip Score (HHS) and the Visual Analogue Scale (VAS) for pain. The most recent radiographs were compared to the initial postoperative radiographs and reviewed for any signs of implant migration, periprosthetic radiolucency, or screw breakage [24,25]. The position of the center of rotation was evaluated pre- and postoperatively and its distance from the anatomic center of rotation (aCOR) was measured in mm. The aCOR was determined using Pierchon’s method [26]. 

Statistical analyses were performed using Sigmaplot 13.0 (Systat Software Inc., San Jose, CA, USA). The Mann–Whitney U test was used to test for significant differences between two groups. The one-way ANOVA on ranks was used to test for significant differences between three or more groups. Cumulative implant survivorship and revision-free survivorship were determined using Kaplan–Meier analysis. The level of significance was set at *p* ≤ 0.05. 

## 3. Results

**Figure 6:**
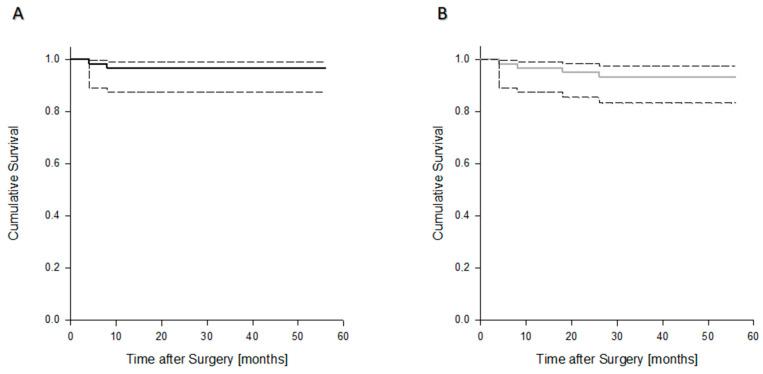
Kaplan–Meier curves demonstrating estimated overall implant survival (**A**) and revision-free survival (**B**).

In our study, the overall revision rate was 6.6% (n = 4). One patient had to undergo a two-stage revision due to deep chronic periprosthetic infection eight months postoperatively. In another patient, the acetabular implant had to be revised due to aseptic loosening four months postoperatively. Therefore, Kaplan–Meier analysis revealed an estimated five-year implant survival of 97% (CI 87–99%). The estimated five-year survival with revision for any cause, on the other hand, was 93% (CI 83–98%) as two additional patients were revised without exchanging the acetabular component (Figure 6). In one patient, recalcitrant groin pain made it necessary to release the psoas tendon arthroscopically. In another one, acute periprosthetic infection rendered it necessary to perform a thorough debridement, irrigation, and exchange of the mobile components. The infection rate in our study was, therefore, 3.3% (n = 2).

Five additional patients suffered from complications that were not treated surgically, resulting in an overall complication rate of 15% (n = 9). One patient suffered from temporary femoral nerve palsy, the symptoms of which resolved completely within six months after surgery. Another patient whose femoral stem was revised to a modular, tapered, uncemented stem sustained a femoral periprosthetic fissure that was recognized postoperatively and successfully treated conservatively by protected weight bearing for a period of six weeks. In another case, there was an intraoperative fracture of the tip of the greater trochanter that was treated conservatively as the displacement was only minimal. The dislocation rate in our study was 3.3% (n = 2). One dislocation occurred five days postoperatively and was successfully treated by closed reduction with otherwise uneventful postoperative rehabilitation and follow-up. Another patient with previously undiagnosed borderline personality suffered from recurrent posterior hip dislocations inflicted by self-aggressive behavior. She was treated repeatedly with closed reduction and she permanently had to wear an anti-dislocation hip brace. 

**Figure 7:**
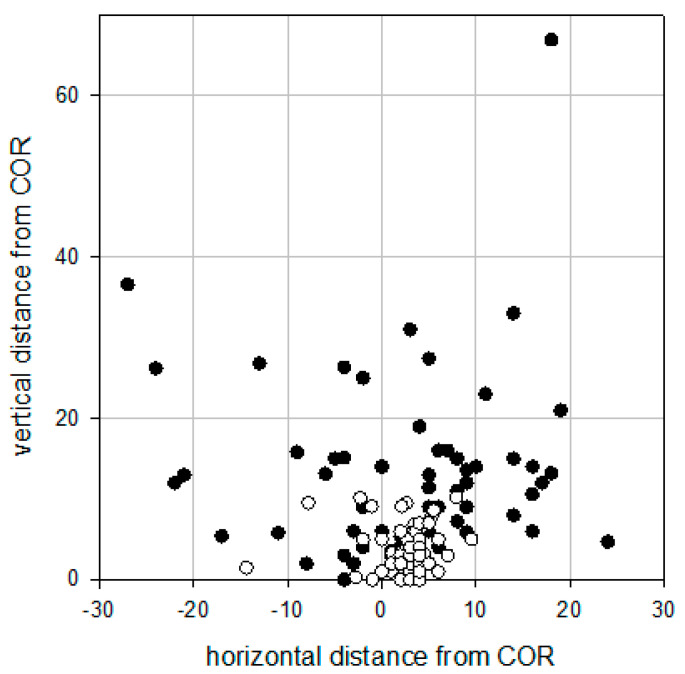
Scatter plot demonstrating the location of the center of rotation pre- (black dots) and postoperatively (white dots). The point of origin (0/0 mm) represents the aCOR.

Radiographic analyses at the latest follow-up revealed well-fixed acetabular components without any signs of migration or screw breakage in all hips that did not undergo acetabular re-revision. In two hips, there were radiolucency lines of more than 2 mm in all three zones but patients were both asymptomatic. Preoperatively, 84% (n = 51) of all the hips’ centers of rotations were located more than 5 mm away from the aCOR. Postoperatively, only 36% (n = 22) were located more than 5 mm away from the aCOR (Figure 7). The median horizontal distance from the aCOR was 7 mm (0–27) preoperatively and 3 mm (0–14) postoperatively (*p* < 0.001). The median vertical distance from the aCOR was 12 mm (0–67) preoperatively and 3 mm (0–10) postoperatively (*p* < 0.001). 

The median HHS and VAS improved significantly from 35 (2–66) and 7 (2–10) preoperatively to 86 (38–100) and 2 (0–6) postoperatively (*p* < 0.001). The median postoperative HHS was significantly higher in patients with no previous revision compared to patients with at least one previous revision (86 vs. 77; *p* = 0.048). There was no statistically significant difference in the median HHS of hips in which the center of rotation was reconstructed within a distance of ≤5 mm from the aCOR and of those in which not (86 vs. 79, *p* = 0.05). The differences between the median postoperative HHS of hips with Paprosky type IIB, IIC, IIIA, or IIIB bone defects were greater than it was expected by chance. The ANOVA on ranks revealed a statistically significant difference (*p* = 0.04). Pairwise multiple comparison procedure (Dunn’s method) revealed a statistically significant difference between the median postoperative HHS of hips with Paprosky type IIC and IIIB bone defects (*p* = 0.048). 

## 4. Discussion

Recent AAHKS surveys have demonstrated that the use and popularity of the DAA for primary total hip arthroplasty is significantly increasing [6,7]. *Nota bene*, the same surveys have shown that only 40% of current DAA performers use the anterior approach for complex primary arthroplasty and only 20% for revision cases. A close look into the literature reveals a similar trend. While the number of studies describing the results after DAA primary hip arthroplasty is rapidly increasing, there is a lack of literature on the use of the DAA for revision arthroplasty [11,12,14,15,27,28,29,30,31,32,33,34]. Whether the lack of available literature and, therefore, the lack of experience within the surgical community causes surgeons to refrain from revising patients via the DAA or whether the DAA is rather not suitable for revision arthroplasty has so far remained largely unanswered. 

Multiple clinical studies have shown that the DAA is able to provide an excellent overview about the acetabulum and the surrounding osseous pelvic structures, which explains the high accuracy of acetabular component placement that can be achieved when using this approach [35]. The pearls and pitfalls of the DAA have been discussed previously at large and it seems that the DAA is associated with lower dislocation rates when compared to posterior approaches and a reduced rate of Trendelenburg positive patients when compared to lateral approaches [2,3,36]. As we know from the literature, revision hip arthroplasty can result in soft tissue defects and particularly lesions of the abductor apparatus, increasing the risk for dislocation and insufficient muscular stabilization of the pelvis during standing. A priori, this makes the DAA an ideal approach for acetabular component revision—especially if the DAA has already been used for the index operation [12,13,37]. On the other hand, there is also data demonstrating that the DAA is associated with a steeper learning curve, a higher perioperative complication rate, and, putatively, with a higher infection rate when compared to other approaches [3,10,36,38]. In the revision setting, this would compromise outcomes. 

The present study was performed in order to evaluate outcomes and revision rates after aseptic acetabular revision through the DAA using an asymmetric acetabular component with optional intra- and extramedullary fixation. The rationale behind the use of this particular implant system has been described previously [21,22]. After a mean follow-up of 40 months, 6.6% of our patients had to be re-revised. This is in line with the few previously published studies available that describe the outcomes after DAA revision arthroplasty. Baba and co-workers reported on the outcomes of patients whose acetabular components were revised through the DAA using Kerboull-type acetabular reinforcement rings. In his series of 21 patients, the overall revision rate was 4.5% after a mean follow-up of 3.8 years [27]. Thaler et al. analyzed outcomes of 64 patients revised through the DAA using the GAPII reconstruction ring. After a mean follow-up of 2.3 years, the overall revision rate was 9.4% [14]. In another study by Horsthemke et al., the overall revision rate was 10.5% after a mean follow-up of 5.4 years. In his series of 48 patients, DAA revision arthroplasty was performed using different revision systems including hemispherical press fit cups with unipolar bearings, cemented dual-mobility cups, or Burch–Schneider reconstruction cages in combination with cemented dual mobility components [30]. These studies describe in detail the types of bone defects encountered intraoperatively, which gives the reader an impression about the complexity of the operative procedures performed [14,27,30]. Earlier studies also report low overall revision rates, but no information about the implant revision strategy or the underlying type of bone defect can be found [31,32]. 

The literature suggests that primary hip arthroplasty via the DAA is associated with low dislocation rates—at least when compared to posterior approaches [3,39]. However, there is only little data about dislocation rates after DAA revision arthroplasty. In our study, 3.3% of the hips dislocated postoperatively. Of note, no constrained liners or dual-mobility cups were used in our series and index operations were performed through different approaches. Cogan and co-workers analyzed outcomes in a series of 61 DAA acetabular component revisions after excluding patients treated with dual-mobility cups or constrained liners and found a dislocation rate of 6.6% [28]. Other studies analyzing the outcome of DAA revision arthroplasty found dislocation rates of 0–4.6% [14,27,29,30,31,32]. In summary, dislocation rates after DAA revision arthroplasty are higher than those observed for primary DAA arthroplasty but similar to those observed for revisions through other approaches [40]. Taking this into consideration, it is likely that in the revision setting the number of previous revisions, the head diameter, and the polarity or constraint of the articulating bearing influences the risk for dislocation to a higher degree than the type of approach [28,41]. 

In the literature, it is still being discussed whether the DAA is associated with a higher infection rate when compared to other approaches or not. Aggarwal et al. analyzed infection rates of 1985 DAA procedures and 4101 non-DAA procedures performed at a single institution between 2013 and 2016. After accounting for co-variates, they found a 2.2 higher likelihood for the development of a periprosthetic joint infection in the DAA group [10]. On the other hand, recent data from the Australian Orthopaedic Association National Joint Replacement Registry demonstrated that after adjustment of hazard ratios for age, gender, ASA score, and BMI, there was a lower rate of revision for infection for the DAA group (n = 32.086) compared to the posterior approach group (n = 65.791) [39]. The literature on infection rates in DAA revision arthroplasty is scarce. The few studies available report on infection rates between 0 to 4.7% [14,27,29,30,32]. In our study, two patients (3.3%) were diagnosed with periprosthetic infection. These findings are similar to infection rates that can be found in the literature for other surgical approaches used in the revision setting. Again, it is likely that—as long as the surgeon is familiar with the approach used – the number of previous revisions and patient-inherent characteristics such as co-morbidities, age, BMI, and body morphology, inter alia, are more important risk factors for the development of a periprosthetic infection than the type of surgical approach chosen [41,42]. Recently, Thaler et al. demonstrated that the DAA might even be safe to treat periprosthetic infection in two-stage revision arthroplasty [15]. 

## 5. Conclusions

We acknowledge that categorical conclusions cannot be drawn from the results of our study due to its retrospective design, its relatively small patient cohort, and due to the fact that there was no control group. However, our study demonstrates that revision arthroplasty of the acetabular component through the DAA using an asymmetric acetabular component with optional intra- and extramedullary iliac fixation is safe and reliable, resulting in good midterm outcomes. No complications related to the approach or the described implant system have been recorded. As with any other approach, the DAA has its pearls and pitfalls but our results demonstrate that the DAA is indeed suitable for acetabular revision arthroplasty—as long as the implant and the instruments used can safely be handled through the DAA and as long as the surgeon is familiar with the approach.
